# Supplementary material for: Architecture of the ring formed by the tubulin homologue FtsZ in bacterial cell division
Source: eLife. 2014 Dec 9;3:e04601. doi: 10.7554/eLife.04601 (PMC4383033; doi:10.7554/eLife.04601)
Supplement: Supplementary file 1. — (A) Plasmids used in this study. (B) Exact protein sequences of modified E. coli FtsZ proteins used for in vivo tomography experiments. DOI: http://dx.doi.org/10.7554/eLife.04601.028 [file elife04601s002.docx]

**Supplementary File**

**1A. List of plasmids used in this study.**

| Name | Description | Resistance | Source |
| --- | --- | --- | --- |
| pMAC382 | pBAD24::GFP(mut2)::icsA(1-104) | Amp | (Charles et al., 2001) |
| pMZ119 | pMAC382, pBAD::ftsZ::mts^minD^ | Amp | This work |
| pMZ120 | pMAC382, pBAD::ftsZ(D212A) | Amp | This work |
| pMZ124 | pMZ120, pBAD::ftsZ(D212A)::ftsA | Amp | This work |
| pMZ125 | pMZ120, pBAD::ftsZ<Q-rich>(D212A) | Amp | This work |
| pMZ127 | pMZ120, pBAD::ftsZ(Δ321-358, D212A) | Amp | This work |
| pMZ129 | pMZ120, pBAD::ftsZ(1-366, D212A) | Amp | This work |
| pSZ7 | pTXB1::Tm-ftsZ | Amp | (Szwedziak et al., 2012) |
| pSZ8 | pTXB1::Tm-ftsA | Amp | (Szwedziak et al., 2012) |
| pSZ78 | pMZ124, pBAD::Tm-ftsZ::Tm-ftsA | Amp | This work |
|  |  |  |  |

Amp: ampicillin; Cml: chloramphenicol; Kan: kanamycin.

Charles, M., Perez, M., Kobil, J. H., and Goldberg, M. B. (2001). Polar targeting of *Shigella* virulence factor IcsA in Enterobacteriacae and *Vibrio.* Proc Narl Acad Sci USA *98*, 9871-9876.

Szwedziak, P., Wang, Q., Freund, S. M., and Löwe, J. (2012). FtsA forms actin-like protofilaments. EMBO J. *31*, 2249-2260.

**1B. Protein sequences.** Please also consult Figure 1–figure supplement 5 for a schematic.

**FtsZ(D212A, Q-rich) from plasmid pMZ125**

(Q-rich: underlined and in blue, corresponds to amino acids 130 – 325 from *E. coli* FtsN protein)

MFEPMELTNDAVIKVIGVGGGGGNAVEHMVRERIEGVEFFAVNTDAQALRKTAVGQTIQIGSGITKGLGAGANPEVGRNAADEDRDALRAALEGADMVFIAAGMGGGTGTGAAPVVAEVAKDLGILTVAVVTKPFNFEGKKRMAFAEQGITELSKHVDSLITIPNDKLLKVLGRGISLLDAFGAANDVLKGAVQGIAELITRPGLMNVDFAAVRTVMSEMGYAMMGSGVASGEDRAEEAAEMAISSPLLEDIDLSGARGVLVNITAGFDLRLDEFETVGNTIRAFASDNATVVIGTSLDPDMNDELRVTVVATGIGMDKRPEITLVTNKQVQQPVMDRYQQHGMAPLTQEQKPVAKVVNDNAPQTAKERQQPTQLVEVPWNEQTPEQRQQTLQRQRQAQQLAEQQRLAQQSRTTEQSWQQQTRTSQAAPVQAQPRQSKPASSQQPYQDLLQTPAHTTAQSKPQQPDYLDIPAFLRKQAD

**FtsZ-mts from plasmid pMZ119**

(mts^minD^: underlined and in blue, corresponds to the C-terminal 16 amino acids of *E. coli* MinD)

MFEPMELTNDAVIKVIGVGGGGGNAVEHMVRERIEGVEFFAVNTDAQALRKTAVGQTIQIGSGITKGLGAGANPEVGRNAADEDRDALRAALEGADMVFIAAGMGGGTGTGAAPVVAEVAKDLGILTVAVVTKPFNFEGKKRMAFAEQGITELSKHVDSLITIPNDKLLKVLGRGISLLDAFGAANDVLKGAVQGIAELITRPGLMNVDFADVRTVMSEMGYAMMGSGVASGEDRAEEAAEMAISSPLLEDIDLSGARGVLVNITAGFDLRLDEFETVGNTIRAFASDNATVVIGTSLDPDMNDELRVTVVATGIGMDKRPEITLVTNKQVQQPVMDRYQQHGMAPLTQEQKPVAKVVNDNAPQTAFIEEEKKGFLKRLFGG

**FtsZ(ΔC17, D212A) from plasmid pMZ129**

MFEPMELTNDAVIKVIGVGGGGGNAVEHMVRERIEGVEFFAVNTDAQALRKTAVGQTIQIGSGITKGLGAGANPEVGRNAADEDRDALRAALEGADMVFIAAGMGGGTGTGAAPVVAEVAKDLGILTVAVVTKPFNFEGKKRMAFAEQGITELSKHVDSLITIPNDKLLKVLGRGISLLDAFGAANDVLKGAVQGIAELITRPGLMNVDFAAVRTVMSEMGYAMMGSGVASGEDRAEEAAEMAISSPLLEDIDLSGARGVLVNITAGFDLRLDEFETVGNTIRAFASDNATVVIGTSLDPDMNDELRVTVVATGIGMDKRPEITLVTNKQVQQPVMDRYQQHGMAPLTQEQKPVAKVVNDNAPQTA

**FtsZ(Δ321-358, D212A) from plasmid pMZ127**

MFEPMELTNDAVIKVIGVGGGGGNAVEHMVRERIEGVEFFAVNTDAQALRKTAVGQTIQIGSGITKGLGAGANPEVGRNAADEDRDALRAALEGADMVFIAAGMGGGTGTGAAPVVAEVAKDLGILTVAVVTKPFNFEGKKRMAFAEQGITELSKHVDSLITIPNDKLLKVLGRGISLLDAFGAANDVLKGAVQGIAELITRPGLMNVDFAAVRTVMSEMGYAMMGSGVASGEDRAEEAAEMAISSPLLEDIDLSGARGVLVNITAGFDLRLDEFETVGNTIRAFASDNATVVIGTSLDPDMNDELRVTVVATGIGMDKRPDYLDIPAFLRKQAD
